# Factors associated with self-harm in patients with substance use disorders who died by suicide: national hybrid questionnaire registry study

**DOI:** 10.1192/bjp.2025.22

**Published:** 2026-03

**Authors:** Martin Ø. Myhre, Fredrik A. Walby, Ole Klungsøyr, Jørgen G. Bramness, Lars Mehlum

**Affiliations:** National Centre for Suicide Research and Prevention, Institute for Clinical Medicine, University of Oslo, Oslo, Norway; Department of Research and Innovation, Division of Mental Health, Oslo University Hospital, Oslo, Norway; Section for Clinical Addiction Research (RusForsk), Oslo University Hospital, Oslo, Norway; Department of Alcohol, Tobacco and Drugs, The Norwegian Institute of Public Health, Oslo, Norway; Institute of Clinical Medicine, UiT The Arctic University of Norway, Tromsø, Norway; Norwegian National Competency Centre for Drug Abuse and Mental Illness, Brumunddal, Norway

**Keywords:** Suicide, substance use disorders, self-harm, register–survey linkage, machine learning methods

## Abstract

**Background:**

Self-harm, self-poisoning or self-injury, irrespective of the motivation, is a central risk factor for suicide. Still, there is limited knowledge of self-harm among patients with substance use disorders (SUDs) who die by suicide.

**Aims:**

We aimed to describe the prevalence of a history of self-harm and identify the factors associated with self-harm, comparing individuals who died by suicide with and without SUDs.

**Method:**

We used data from the Norwegian Surveillance System for Suicide in Mental Health and Substance Use Services, which is based on a national linkage between the Norwegian Cause of Death Registry and the Norwegian Patient Registry, to identify individuals who died by suicide within 1 year after last contact with mental health or substance use services (*n* = 1140). A questionnaire was retrieved for 1041 (91.3%) of these individuals. We used least absolute shrinkage and selection operator (LASSO) regression to select variables and compared patients with and without SUDs. Conditional selective inference was used to improve 90% confidence intervals and *p*-values.

**Results:**

The prevalence of self-harm was 55% in patients with SUDs and 52.6% in patients without SUDs. Suicidal ideation (odds ratio 2.98 (95% CI 1.74–5.10)) emerged as a factor shared with patients without SUDs, while personality disorders (odds ratio 1.96 (1.12–3.40)) and a history of violence (odds ratio 1.86 (1.20–2.87)) were unique factors for patients with SUDs.

**Conclusions:**

A history of self-harm is prevalent in patients with SUDs who die by suicide and is associated with suicidal ideation, a history of violence and personality disorders in patients with SUDs.

Self-harm is an act of self-injury or self-poisoning, irrespective of the motivation.^1^ Having a history of self-harm is a central risk factor for suicide.^2,3^ Other factors may also increase the risk of suicide, but the importance of these may be different between those with or without a history of self-harm. For example, patients who died by suicide after contact with mental health services (MHSs) with previous self-harm differ from those without^4^ in their more extended duration of healthcare contact; they die closer in time to the last contact, and they have a higher clinician-rated suicide risk.

Patients with substance use disorders (SUDs) have an increased risk of suicide.^5^ However, only a few studies have described the risk factors for suicide in patients with SUDs. The risk factors identified are the severity of the SUD, aggression or impulsivity, negative affect, active SUD, interpersonal stress and depressive symptoms.^6^ Currently, evidence-based suicide prevention strategies are insufficiently developed,^7^ but more knowledge on what characterises patients who have a history of self-harm, a central risk factor for suicide in patients with SUDs, compared to those who have not, could contribute to preventive interventions.

## Self-harm in patients with substance use disorders

The risk of self-harm is increased in patients with SUD with comorbid mental disorders.^8^ Moreover, in patients with a history of self-harm, SUD is a predictor for subsequent suicide death.^9^ We have identified only one previous study that examined self-harm and its association with suicide in patients with SUDs,^10^ where self-harm was an important risk factor for suicide in women but not in men. All this taken together, the prevalence of self-harm in patients with SUDs, the factors associated with self-harm in this group and self-harm as a risk factor for suicide are poorly understood. Improved knowledge about these factors is necessary to increase our understanding and improve the clinical management of self-harm in patients with SUDs and, ultimately, how we prevent suicide in this group. Moreover, patients with SUDs constitute a heterogeneous group; thus, examining possible differences between patients with alcohol use disorders (AUDs) and drug use disorders (DUDs) could provide important insights.

## Study aims

We aimed to study the prevalence of a history of self-harm in patients in contact with MHSs or substance use services (SUSs) who died by suicide within 1 year of last contact, comparing patients with and without SUDs. We also wanted to identify the factors associated with a history of self-harm in patients with SUDs compared to patients without SUDs. In addition, we conducted a subgroup analysis examining self-harm in patients with AUD and DUD in the SUD group using the same approach.

## Method

### Sample

Information on all Norwegian residents who died by suicide (ICD-10 codes:^11^ X60–X84; Y10–Y35; Y870; Y872) between 1 January 2018 and 31 December 2021 was retrieved from the Norwegian Cause of Death Registry (NCDR) (*n* = 2693) and linked with the Norwegian Patient Registry (NPR). All cases of suicide in people above 18 years old who were registered with at least one contact with MHSs or SUSs in the year before their suicide were included (*n* = 1140). The study sample was separated into people with SUDs (*n* = 358) and without SUDs (*n* = 683). The SUD group was also split between patients with AUD (*n* = 145) and DUD (*n* = 213). See Supplementary material 1 for further information.

### Design

The study was a national hybrid registry questionnaire study, combining historical prospective data from national administrative health registers with a retrospective clinical questionnaire.

### Data collection

We used data from the Norwegian Surveillance System for Suicide in Mental Health and Substance Use Services (NoSS), which gathers information on all individuals who died by suicide who had contact with MHSs or SUSs within 1 year before the suicide.^12^ The NoSS uses a registry linkage between the NCDR and the NPR. A questionnaire is then linked with the registry linkage through a pseudo-ID produced by the 11-digit personal ID assigned to each Norwegian citizen. The pseudo-ID is generated through a one-way hashing algorithm.^12^ A registry linkage between the NCDR and the NPR defines the sample as described above, retrieving registry data from the NCDR and the NPR for the sample.

A questionnaire, filled in by clinicians who had the last contact with the patients, is gathered retrospectively after a patient has died by suicide. This questionnaire is an adaptation of a similar system developed by the National Confidential Inquiry into Suicide and Safety in Mental Health in the UK^13^ and was used with approval from them. The questionnaire is designed to describe the circumstances around suicide and the characteristics and treatment of the patient who died by suicide. Clinicians can report the questionnaire both directly after a patient dies by suicide or prompted by the NoSS after the annual registry linkage is retrieved. A full description of the data collection system is available elsewhere.^12^

The NCDR is a national health registry containing data on all deaths and their causes in Norway, and it has high coverage and good data quality.^14^ The NPR is a national health registry containing information about contacts with specialised health services in Norway. For the whole period between 2018 and 2021, the coverage of the NPR was excellent, with good data quality.^15^ The coverage of the questionnaire was also good, with a total response rate of 91.3%.

### Variable of interest

The variable of interest was a history of self-harm episodes measured through the questionnaire item ‘Did the patient have a history of deliberate self-harm?’ coded as ‘No’, ‘Yes, during the last three months’, ‘Yes, more than three months ago’, ‘Yes, both during the last three months, and more than three months ago’, ‘Yes, but details are unknown’ and ‘Unknown’. The index time was from the last contact with services. All the ‘Yes’ responses were recoded as ‘Yes’, whereas other responses were coded as ‘No’. ‘Unknown’ responses (*n* = 113) were treated as missing data and were excluded from further analyses.

### Factors possibly associated with a history of self-harm

We identified whether the person had any SUD (F10–F16; F18; F19) registered in the NPR within 1 year before suicide. People with a SUD were further divided into people with AUD (F10) and DUD (F11–F16; F18; F19) based on the SUD last registered in the NPR.

All available variables that described the patient and their treatment were included in the questionnaire, supplemented with registry data. They are described in Supplementary Material 3. The questionnaire included information about sociodemographic factors, history of exposure to adverse life events, violence or abuse, psychiatric history, previous and current treatment and the last contact. From the NCDR, gender and age were retrieved. Age was transformed into a categorical variable with four levels based on the quantiles of the total study sample. From the NPR, ICD-10 diagnoses for mental health and SUDs within the previous year, service contact within 1 year before suicide, the last in-patient admission and the last contact with MHSs or SUSs were retrieved. All variables were categorical and recoded into binary dummy variables. A full description of the variables and the reference level for the variables is given in Supplementary Table 2. Variables that occurred at very low frequencies, defined as less than ten unique values or less than 5% occurrence, were removed from the variable set eligible for selection (*k* = 21).

### Analysis

We used a sequential regression procedure to identify the factors associated with self-harm. First, we used machine learning to build models of the variable set that best predicted self-harm. Then, we used post-selection inference to provide improved confidence intervals and *p*-values for the selected variables. Traditional approaches where regression models are built after data exploration are susceptible to type I errors. The advantage of this procedure is that it accounts for the prior exploration of data and thus yields improved confidence intervals and *p*-values.

The prediction models were built with the least absolute shrinkage and selection operator (LASSO) procedure with the binomial model.^16,17^ Self-harm was the outcome. The LASSO is a penalised algorithm that uses an L^1^ penalty to regularise the regression models. The algorithm shrinks the coefficients towards zero, thereby conducting variable selection.^18^ The LASSO is useful since it selects sparse models from large variable sets. Models were built with ten-fold cross-validation to assess model performance and reduce the probability of overfitting. Models were fitted over a grid of values of the regularisation parameter lambda. The most regularised model, represented by the lambda with a cross-validated error within one standard error of the minimum, was used.^16^ The area under the curve (AUC) was used as a performance measure to select the best model and measure the model’s performance in predicting the outcome.

While the LASSO can conduct variable selection, it does not provide confidence intervals or *p*-values. We used the post-selection inference method of conditional selective inference to provide improved confidence intervals and *p*-values of the coefficients selected by the LASSO.^19^ The relevant one-step selective inference was used here to estimate partial regression coefficients.^20^ Conditional selective inference provides inference on estimates selected by the LASSO by conditioning them on the prior variable selection, and yields estimates like data-splitting strategies. We used a 90% confidence level as this provided adequate coverage of the partial regression models containing the set of variables selected by the LASSO procedure,^20^ as used here. This approach contributes to identifying factors containing a strong signal for predicting self-harm.

As described above, the analysis was stratified by whether individuals were registered with a SUD in the past year or not to ensure that differences would be discovered. We also conducted a subgroup analysis, separating the patients with SUDs into patients with AUD and DUD, utilising an identical analytic strategy within all groups. The analysis was performed in R version 4.2.2 for Windows (R Foundation for Statistical Computing, Vienna, Austria; see https://cran.r-project.org/), using the packages *glmnet* (for Windows, developed by Friedman et al; see https://cran.r-project.org/web/packages/glmnet/) ^16^ for the LASSO regression, the *selectiveInference* package (for Windows, developed by Tibshirani et al; see https://CRAN.R-project.org/package=selectiveInference for post-selection inference and *ggplot2* (for Windows, developed by Wickham; see https://ggplot2.tidyverse.org)^21^ for visualisation.

### Ethical approval

Since this was a retrospective study of individuals who died by suicide, informed consent was impossible to obtain. An approved external data manager in the NPR handled personal IDs, and the authors have not had access to the 11-digit personal IDs or any other direct person-identifying information. The Norwegian Directorate of Health granted exemption from patient confidentiality for the NoSS (ref: 16/27835-12). The study was approved by the Regional Committee for Medical and Health Research Ethics South-East Norway (ref: 32494).

## Results

Having a history of self-harm was highly prevalent in both patients with (55.0%) and without SUDs (52.6%) who died by suicide. In the subgroup analysis of patients with SUDs, patients with AUD and DUD had comparable self-harm prevalences (55.9 and 54.5%, respectively). Missing responses on the self-harm item ranged from 9.2% in patients without SUDs to 15.5% in patients with DUDs. These people with missing data on self-harm were excluded. Additional characteristics of the sample are provided in Table 1.

**Table 1:** Prevalence of a history of self-harm in patients who died by suicide with and without substance use disorders

Disorder	Self-harm	*n* (%)	Males (%)	Age Mean (s.d.)
Substance use disorders	No self-harm	111 (31.0)	81.1	42.9 (13.6)
	Self-harm	197 (55.0)	66.0	40.0 (13.0)
	Missing	50 (14.0)	72.0	42.4 (13.7)
Alcohol use disorder	No self-harm	47 (32.4)	85.1	48.3 (12.1)
	Self-harm	81 (55.9)	59.3	42.9 (12.8)
	Missing	17 (11.7)	76.5	49.1 (14.3)
Drug use disorder	No self-harm	64 (30.0)	78.1	38.9 (13.4)
	Self-harm	116 (54.5)	70.7	38.1 (12.9)
	Missing	33 (15.5)	69.7	38.9 (12.2)
No substance use disorders	No self-harm	261 (38.2)	71.6	47.7 (16.4)
	Self-harm	359 (52.6)	49.9	45.4 (16.8)
	Missing	63 (9.2)	65.1	45.4 (15.1)

### Patients with substance use disorders compared with other patients

In patients with SUDs (*n =* 308), the LASSO model contained seven factors with an AUC of 76.3%. In contrast, the LASSO model in patients without SUDs (*n* = 620) included 49 factors with an AUC of 82.7%. In both models, the sensitivity was excellent, while the specificity was poor (Table 2). The cross-validation and receiver operating characteristic (ROC) curves are provided in Supplementary Materials 4 and 5, respectively.

**Table 2:** Model’s performance of predicting self-harm in patients who died by suicide in different strata

Disorder	*n*	Log (λ)	Non-zero variables	Sensitivity	Specificity	AUC
Patients with substance use disorders	308	1.348	7	1.000	0.000	0.763
Patients without substance use disorders	620	1.098	49	0.811	0.636	0.827
Patients with alcohol use disorder	128	1.045	7	0.963	0.636	0.885
Patients with drug use disorder	180	1.165	7	1.000	0.000	0.822

AUC, area under the curve.

**Fig. 1:**
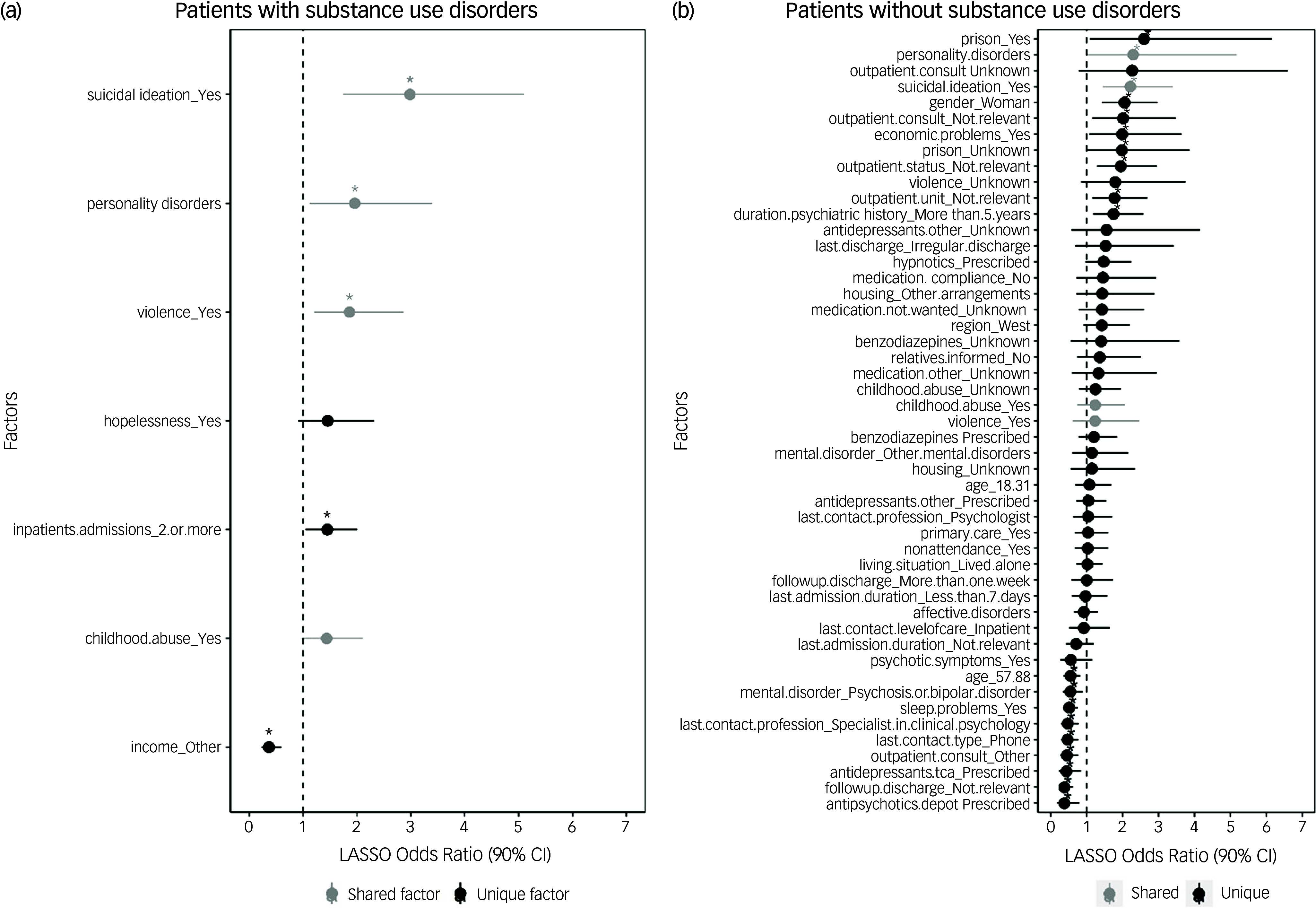
Odds ratios of the features selected by least absolute shrinkage and selection operator (LASSO) regression models with conditional post-selection 95% confidence intervals in patients (a) with and (b) without substance use disorders. Grey points and lines indicate shared factors, while black factors are unique. Significant variables are illustrated with an asterisk.

The bivariate distribution of the factors included is shown in Supplementary Material 4. The estimates of the association between the factors selected by the LASSO models and self-harm in patients with and without SUDs are illustrated in Fig. 1 and Supplementary Table 6. In patients with SUDs, four factors with increased odds emerged: having suicidal ideation at the last treatment contact (odds ratio 2.98 (conditional 95% CI 1.74–5.10); *p* = 0.003); having a personality disorder (odds ratio 1.96 (1.12–3.40); *p* = 0.047); having a lifetime history of violent behaviour (odds ratio 1.86 (1.20–2.87); *p* = 0.044); and more than two admissions in the past year (odds ratio 1.45 (1.04–2.00), *p* = 0.062). In patients without SUDs, six factors associated with self-harm emerged. The top three were suicidal ideation at the last contact (odds ratio 2.21 (1.44–3.41); *p* = 0.039), female gender (odds ratio 2.05 (1.42–2.97); *p* = 0.039) and not having had an out-patient consultation (odds ratio 2.01 (1.16–3.48); *p* = 0.037).

### Patients with alcohol or drug use disorders

Model performance was better in the subgroup analysis for patients with AUD and DUD. In patients with AUD (*n* = 128), the LASSO model had seven factors with an AUC of 88.5%. In patients with DUD (*n* = 180), the LASSO model contained seven factors with an AUC of 82.2%. As in the primary analyses, the sensitivity was excellent, but the specificity was poor (Table 2).

**Fig. 2:**
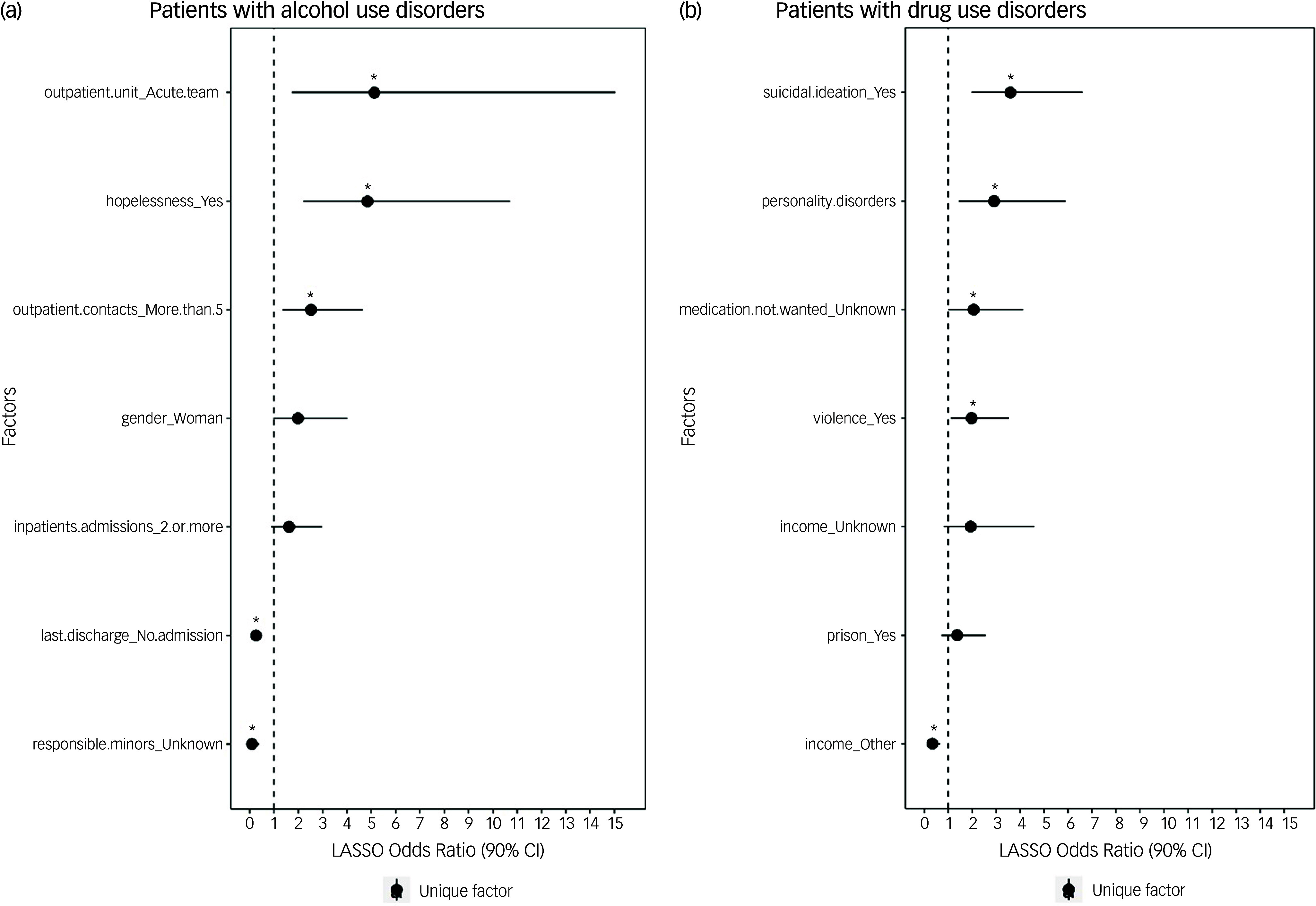
Odds ratios of the features selected by least absolute shrinkage and selection operator (LASSO) regression models with conditional post-selection 95% confidence intervals in patients with (a) alcohol and (b) drug use disorders. Significant variables are illustrated with an asterisk.

The selected factors and their association with self-harm in patients with AUD and DUD are illustrated in Fig. 2, with estimates provided in Supplementary material 7. For patients with AUD, the factors emerging were having the last out-patient contact with an acute team compared to a general out-patient clinic (odds ratio 5.12 (1.73–15.03); *p* = 0.020), hopelessness at the last contact (odds ratio 4.84 (2.18–10.69); *p* = 0.004) and having more than five out-patient contacts in the past year (odds ratio 2.51 (1.35–4.66); *p* = 0.014). For patients with DUD, having suicidal ideation at the previous contact (odds ratio 3.59 (1.96–6.60); *p* = 0.004), having a personality disorder (odds ratio 2.91 (1.44–5.89); *p* = 0.030) and having a history of violence (odds ratio 1.98 (1.10–3.53); *p* = 0.054) emerged as factors that increased the odds of self-harm.

## Discussion

In this study of patients who died by suicide, we found a similar prevalence of self-harm in patients with SUDs compared to patients without SUDs. Using a data-driven approach with improved confidence intervals, we identified which factors were most strongly associated with self-harm in patients with and without SUDs. Whereas suicidal ideation at the last treatment contact was the only shared factor, a history of violent behaviour and a diagnosis of personality disorder were uniquely related with self-harm in patients with SUDs. The factors uniquely associated with self-harm in patients without SUDs were female gender and not having had any out-patient consultations. When separating patients with AUD and DUD in the subgroup analysis, all factors that emerged as significant were unique. Hopelessness, having had acute team contact, more than five out-patient contacts and no hospital admission were related to self-harm in patients with AUD. In contrast, suicidal ideation and a diagnosis of personality disorder were related to self-harm in patients with DUDs.

### Factors associated with a history of self-harm

Not surprisingly, suicidal ideation at the last contact was strongly associated with a history of self-harm. Although this factor was shared between all patients who died by suicide, it was especially prominent in patients with DUD. Suicidal ideation is a well-known risk factor for both self-harm and suicide death.^22^ Still, information on this factor is commonly unavailable in routine data such as register data or electronic health records in MHSs or SUSs often used in previous studies,^10,23,24^ although some reports have been published on suicidal ideation based on data from emergency departments.^25,26^

In patients with SUDs, a diagnosis of personality disorder and a history of violent behaviour emerged as factors associated with self-harm. This is not surprising since violence often arises from a combination of aggression and lack of impulse control, which are factors found to be distally associated with self-harm in patients with SUDs.^6^ A history of self-harm and violence could be associated both directly and through a shared vulnerability for emotion regulation problems. We found some support for the latter through an association with personality disorders. A history of self-harm is very prevalent in patients with borderline personality disorder^27^ and other cluster B personality disorders, and these disorders are strongly associated with SUDs.^28^ Our findings correspond well with a study from the UK of patients with personality disorders who died by suicide, where a history of self-harm, violence and substance use was more prevalent than in any other diagnoses.^29^

An association between being a woman and self-harm was present in patients without SUDs and in patients with AUD but not in patients with SUDs or DUD. The risk of self-harm is stronger in women, but the suicide risk is higher for men after presenting to services with self-harm.^30–32^ Why this association with gender is not present in patients with SUDs and DUD needs to be further examined. This may also be of importance given the high suicide rates found in women with SUDs.^33^

In patients with AUD, acute team contact, hopelessness, more than five out-patient contacts and no admissions emerged as unique factors. All factors differed from patients with DUD. The strongest factor emerging in patients with AUD was having the last out-patient contact with an acute team. Acute teams in Norway have many similarities with crisis resolution teams in England, and suicidal ideation is prevalent in users of such crisis services.^34^ Since people with AUD frequently are undertreated^35^ and many thus lack connection to services, acute teams could function as points of contact when patients with AUD experience a suicidal crisis. Hopelessness at the last contact also emerged as a factor strongly associated with a history of self-harm in patients with AUD who died by suicide, whereas suicidal ideation was not. Hopelessness is a well-known risk factor for suicide and suicidal behaviours.^36^ We have no information to explain these differences in associations with hopelessness in patients with AUD and with suicidal ideation in patients with DUD, but it could be linked to a higher level of despair in people with AUD, possibly because of failed attempts of seeking help. That self-harm was also associated with more than five out-patient contacts in patients with AUD could support this view. In a previous study,^31^ we found brief contact trajectories to be prevalent in patients with AUD who died by suicide, and the finding of a greater number of out-patient contacts points to more frequent contact being associated with self-harm.

### Clinical implications

Since examination of a history of self-harm is usually a part of the routines for suicide risk assessment in most clinical units,^37^ services will often possess information about this risk factor, which often is limited in registry studies. Our findings suggest that this information is relevant and should be used more proactively in treatment planning and clinical risk management. Given the current lack of knowledge regarding self-harm in patients with SUDs, this study provides some insights into the differential factors associated with self-harm. The association of suicidal ideation, a history of violence and personality disorders with self-harm in patients with SUDs points to a complex patient group probably in need of long-term, intensive and flexible treatment.

### Strengths and limitations

A strength of this study lies in supplementing national registry data with a nationwide clinical questionnaire with high coverage. Most of the factors that emerged as significant were from the questionnaire and not the national registries, illustrating that the information presented here is relevant.^38^ Another strength is using LASSO regression to select the variables and recent advancements in selective inference to provide confidence intervals and *p*-values that account for the cost of data exploration.^19,20^ This enabled us to provide improved confidence intervals and conduct inferences regarding the factors identified and contributed to reducing the number of important factors. The model’s performance was comparable to previous studies,^39^ and was acceptable for patients with SUDs and excellent in the other groups. While building a prognostic predictive model was not this study’s primary aim, the factors’ relevance depends on adequate model performance. As in other studies, the sensitivity was very good, but the specificity was low. Sensitivity being higher than specificity, especially in the group of patients with SUDs and DUD, which is because few factors not being associated with self-harm were selected. The model’s performance also increased when examining less heterogeneous groups, such as patients with AUD or DUD. Likewise, the LASSO selected many variables in the most heterogeneous group containing patients without SUDs to achieve adequate performance.

This study also has important limitations. External validation is needed to assess the replicability and generalisability of the models. The variable selection process was data driven, and the resulting black box models offer a limited explanation of the variables selected, except for the degree of regularisation, which gives the best model performance. Thus, information on why or how the variables were selected is limited. The novel factors identified need replication and further description, such as the association between acute team use and self-harm in patients with AUD. Data-driven models can be a relevant starting point since the LASSO handles high-dimensional data well when theoretical and empirical descriptions are limited. Interactions were not modelled in this study since differential interactions may be present between the groups examined. Comorbid mental or physical disorders are a plausible interaction that could have affected the variables selected in the groups that should be further examined. The factors from the questionnaire are all reported after the patients died by suicide, making the responses prone to recall bias. The outcome included explicit unknown responses, which were excluded from further analysis to focus on the presence or absence of self-harm. The factors from the questionnaire also included unknown responses that were eligible for selection and were difficult to interpret since these factors represent a lack of information from the clinician responding, who may have multiple reasons.

The factors associated with self-harm before suicide differ between patients with SUDs compared to patients without SUDs and between patients with AUD and DUD within the SUD group. A history of self-harm in patients with SUDs has been understudied, and this study has identified factors uniquely associated with self-harm in patients with SUDs and within the SUD group. This has implications for the development of policies for suicide prevention and the clinical care of patients with SUDs who have harmed themselves. To better understand the contribution of the unique factors, further research is needed.

## Supporting information

Myhre et al. supplementary material Myhre et al. supplementary material

## Data Availability

Because of confidentiality restrictions, the data in this study is not openly available.

## Supplementary material

The supplementary material can be found at https://doi.org/10.1192/bjp.2025.22

## Transparency declaration

This study was exploratory and registered as such at the Open Science Framework before the data analysis (https://osf.io/bfgwa).

## Analytic code availability

The analytic code used in this study is available from the Open Science Framework (https://osf.io/35bxy/).
