# Supplementary material for: Factors associated with self-harm in patients with substance use disorders who died by suicide: national hybrid questionnaire registry study
Source: Br J Psychiatry. 2026 Mar;228(3):229–35. doi: 10.1192/bjp.2025.22 (PMC12912876; doi:10.1192/bjp.2025.22)
Supplement: Myhre et al. supplementary material [file S0007125025000224sup001.docx]

**Supplementary materials overview**

[Supplementary material 1. Flowchart of patients in the study](#_Supplementary_1._Flowchart)

[Supplementary material 2. List of predictors included in the models and their reference level](#_Supplementary_2._List)

[Supplementary material 3. Bivariate distribution of the features across the outcome.](#_Supplementary_3._Bivariate)

[Supplementary material 4. Regularization paths for the LASSO regression models](#_Supplementary_4._The)

[Supplementary material 5. ROC curves for the LASSO regression models](#_Supplementary_5._ROC)

[Supplementary material 6. Estimates and confidence intervals for post-selection debiased and bootstrapped models in patients with SUDs and no SUDs](#_Supplementary_6._The)

[Supplementary material 7. Estimates and confidence intervals for post-selection debiased and bootstrapped models in patients with AUD and DUDs](#_Supplementary_7._The)

# **Supplementary 1.** Flowchart of patients in the study.

Suicide (X60-X84; Y10-Y35; Y870; Y872)

(The Cause of Death Registry)

N = 2 693

Contact with services for mental health or substance the last year

(The Norwegian Patient Registry)

N = 1 140 (42.3 %)

N =

Study population

N = 1 140 (42.3 %)

Clinical survey missing

N = 99 (8.7 %)

Clinical survey received

N = 1 041 (91.3 %)

Deliberate self-harm ‘unknown’ excluded

N = 113 (9.9 %)

Drug use disorder (F11-F16; F18-F19)

N = 180 (58.4 %)

No substance use disorder last year (F20-F99)

N = 620 (41.6 %)

Drug use disorder (F11-F16; F18-F19)

(66.8 %)

Substance use disorder last year (F10-F19)

N = 308 (33.2 %)

Alcohol use disorder (F10)

N = 128 (41.6 %)

# **Supplementary 2**. List of predictors included in the models and their reference level

| Variable | Reference level | Factor level 2 | Factor level 3 | Factor level 4 | Factor level 5 | Factor level 6 |
| --- | --- | --- | --- | --- | --- | --- |
| gender | Man | Woman |  |  |  |  |
| age | 32.44 | 18.31 | 45.56 | 57.88 |  |  |
| region | South.East | Middle | North | West |  |  |
| marital | Married.cohabiting | Divorced.separated | Single | Unknown | Widowed |  |
| income | Working | Other | Sick.leave | Unknown | Welfare.benefits |  |
| housing | Own.house.or.apartment | No.fixed.housing | Other.arrangements | Unknown |  |  |
| living.situation | Lived.with.partner | Lived.alone | Lived.with.other.people | Unknown |  |  |
| responsible.minors | No | Unknown | Yes |  |  |  |
| mental.disorder | No.mental.disorder | Depressive.or.anxiety.disorder | Other.mental.disorders | Psychosis.or.bipolar.disorder |  |  |
| charlson.score | X0 | X1.2 | X3. |  |  |  |
| duration.psychiatric.history | X1.5.years | Less.than.1.year | More.than.5.years | No.mental.disorder | Unknown |  |
| violence | No | Unknown | Yes |  |  |  |
| prison | No | Unknown | Yes |  |  |  |
| childhood.abuse | No | Unknown | Yes |  |  |  |
| lifevent | No | Unknown | Yes |  |  |  |
| economic.problems | No | Unknown | Yes |  |  |  |
| suicide.family | No | Unknown | Yes..family | Yes..friends |  |  |
| inpatient.n.cat | No.admission | X1.admission | X2.or.more.admissions |  |  |  |
| outpatient.n.cat | Less.than.5.contacts | More.than.5.contacts | No.outpatient.contact |  |  |  |
| last.admission.duration | More.than.7.days | Less.than.7.days | Not.relevant |  |  |  |
| last.admission.readmission | No | Not.relevant | Yes |  |  |  |
| last.discharge | Discharged.as.planned | Irregular.discharge | No.admission | Other |  |  |
| followup.discharge | Within.one.week | More.than.one.week | No.appointment | Not.relevant | Patient.did.not.want.outpatient.followup | Patient.died.before.first.followup.after.discharge |
| outpatient.status | Current.contact | Ended.contact | Not.relevant | Unknown |  |  |
| outpatient.unit | Outpatient.clinic | Acute.team | Ambulatory.team..including.ACT.FACT. | Not.relevant | Other | Unknown |
| outpatient.consult | Planned.consultation | Emergency.care.consultation | Not.relevant | Other | Unknown |  |
| primary.care | No | Unknown | Yes |  |  |  |
| individual.plan | No | Offered.but.did.not.want | Unknown |  |  |  |
| antipsychotics.per.os | Not.prescribed | Prescribed | Unknown |  |  |  |
| antipsychotics.depot | Not.prescribed | Prescribed | Unknown |  |  |  |
| moodstabilizer | Not.prescribed | Prescribed | Unknown |  |  |  |
| antidepressants.tca | Not.prescribed | Prescribed | Unknown |  |  |  |
| antidepressants.other | Not.prescribed | Prescribed | Unknown |  |  |  |
| benzodiazepines | Not.prescribed | Prescribed | Unknown |  |  |  |
| hypnotics | Not.prescribed | Prescribed | Unknown |  |  |  |
| opiates.other | Not.prescribed | Prescribed | Unknown |  |  |  |
| opiates.substitution | Not.prescribed | Prescribed | Unknown |  |  |  |
| medication.other | Not.prescribed | Prescribed | Unknown |  |  |  |
| medication.not.wanted | No | Not.relevant | Unknown | Yes |  |  |
| medication.compliance | Yes | No | No.prescription | Unknown |  |  |
| medication.sideeffects | No | No.prescription | Unknown | Yes |  |  |
| last.contact.levelofcare | Outpatient | Inpatient |  |  |  |  |
| nonattendance | No | Not.relevant | Unknown | Yes |  |  |
| last.contact.type | Face.to.face | E.mail | Phone | SMS | Unknown |  |
| last.contact.profession | Psychiatrist | MD.under.specialization | Nurse | Other | Psychologist | Specialist.in.clinical.psychology |
| last.contact.primary.contact | Yes | No | No.primary.contact | Unknown |  |  |
| crisisplan | Yes | No | Unknown |  |  |  |
| relatives.informed | Yes | No | Not.relevant | Unknown |  |  |
| anxiety.symptoms | No | Unknown | Yes |  |  |  |
| depressive.symptoms | No | Unknown | Yes |  |  |  |
| deterioration.physical | No | Unknown | Yes |  |  |  |
| psychotic.symptoms | No | Unknown | Yes |  |  |  |
| hostility | No | Unknown | Yes |  |  |  |
| increased.alcohol.use | No | Unknown | Yes |  |  |  |
| increased.drug.use | No | Unknown | Yes |  |  |  |
| hopelessness | No | Unknown | Yes |  |  |  |
| suicidal.ideation | No | Unknown | Yes |  |  |  |
| sleep.problems | No | Unknown | Yes |  |  |  |

# **Supplementary 3**. Bivariate percentwise of self-harm stratified by whether the patients had a substance use disorder or no substance use disorder and alcohol or drug use disorder registered within the last year.

|  | Substance use disorders (*n* = 308) | |  | No substance use disorder (*n* = 620) | |  | Alcohol use disorder (*n* = 128) | |  | Drug use disorder (*n* = 180) | |
| --- | --- | --- | --- | --- | --- | --- | --- | --- | --- | --- | --- |
| variable | No self-harm | Self-harm |  | No self-harm | Self-harm |  | No self-harm | Self-harm |  | No self-harm | Self-harm |
| psychosis.disorders | 16,2 % | 13,7 % |  | 15,7 % | 11,7 % |  | 2,1 % | 6,2 % |  | 26,6 % | 19,0 % |
| affective.disorders | 25,2 % | 32,5 % |  | 51,7 % | 57,7 % |  | 23,4 % | 33,3 % |  | 26,6 % | 31,9 % |
| anxiety.disorders | 29,7 % | 35,5 % |  | 33,3 % | 39,8 % |  | 29,8 % | 38,3 % |  | 29,7 % | 33,6 % |
| personality.disorders | 5,4 % | 21,8 % |  | 4,2 % | 17,5 % |  | 8,5 % | 19,8 % |  | 3,1 % | 23,3 % |
| behavioral.disturbances | 9,0 % | 13,2 % |  | 4,2 % | 4,5 % |  | 4,3 % | 7,4 % |  | 12,5 % | 17,2 % |
| other.mental.disorders | 28,8 % | 35,5 % |  | 48,3 % | 47,9 % |  | 14,9 % | 34,6 % |  | 39,1 % | 36,2 % |
| psychological.treatment | 26,1 % | 21,8 % |  | 18,8 % | 24,0 % |  | 27,7 % | 27,2 % |  | 25,0 % | 18,1 % |
| district.psychiatric.centre | 26,1 % | 25,9 % |  | 44,1 % | 40,9 % |  | 25,5 % | 24,7 % |  | 26,6 % | 26,7 % |
| crisis.services | 4,5 % | 8,6 % |  | 15,3 % | 12,8 % |  | 2,1 % | 13,6 % |  | 6,3 % | 5,2 % |
| ambulatory.team | 11,7 % | 13,2 % |  | 7,7 % | 6,1 % |  | 4,3 % | 12,3 % |  | 17,2 % | 13,8 % |
| sud.treatment | 36,9 % | 33,0 % |  | 0,8 % | 1,1 % |  | 51,1 % | 37,0 % |  | 26,6 % | 30,2 % |
| gender_Woman | 18,9 % | 34,0 % |  | 28,4 % | 50,1 % |  | 14,9 % | 40,7 % |  | 21,9 % | 29,3 % |
| age_18.31 | 26,1 % | 30,5 % |  | 20,3 % | 26,2 % |  | 10,6 % | 24,7 % |  | 37,5 % | 34,5 % |
| age_45.56 | 26,1 % | 21,3 % |  | 24,5 % | 27,0 % |  | 34,0 % | 34,6 % |  | 20,3 % | 12,1 % |
| age_57.88 | 18,0 % | 13,2 % |  | 33,0 % | 24,8 % |  | 25,5 % | 14,8 % |  | 12,5 % | 12,1 % |
| region_Middle | 7,2 % | 7,6 % |  | 14,2 % | 10,6 % |  | 4,3 % | 6,2 % |  | 9,4 % | 8,6 % |
| region_North | 9,9 % | 14,2 % |  | 9,6 % | 11,4 % |  | 10,6 % | 14,8 % |  | 9,4 % | 13,8 % |
| region_West | 21,6 % | 16,2 % |  | 16,1 % | 21,4 % |  | 27,7 % | 14,8 % |  | 17,2 % | 17,2 % |
| marital_Divorced.separated | 21,6 % | 11,7 % |  | 16,5 % | 17,5 % |  | 34,0 % | 18,5 % |  | 12,5 % | 6,9 % |
| marital_Single | 53,2 % | 66,5 % |  | 41,4 % | 50,4 % |  | 38,3 % | 56,8 % |  | 64,1 % | 73,3 % |
| income_Other | 24,3 % | 11,7 % |  | 27,2 % | 22,6 % |  | 23,4 % | 14,8 % |  | 25,0 % | 9,5 % |
| income_Sick.leave | 4,5 % | 3,0 % |  | 16,5 % | 11,7 % |  | 4,3 % | 4,9 % |  | 4,7 % | 1,7 % |
| income_Unknown | 4,5 % | 13,7 % |  | 5,0 % | 8,1 % |  | 8,5 % | 9,9 % |  | 1,6 % | 16,4 % |
| income_Welfare.benefits | 48,6 % | 57,9 % |  | 28,7 % | 40,4 % |  | 38,3 % | 46,9 % |  | 56,3 % | 65,5 % |
| housing_Other.arrangements | 17,1 % | 18,3 % |  | 7,3 % | 12,8 % |  | 8,5 % | 12,3 % |  | 23,4 % | 22,4 % |
| housing_Unknown | 7,2 % | 11,7 % |  | 5,0 % | 11,7 % |  | 10,6 % | 7,4 % |  | 4,7 % | 14,7 % |
| living.situation_Lived.alone | 61,3 % | 62,9 % |  | 45,6 % | 59,3 % |  | 59,6 % | 74,1 % |  | 62,5 % | 55,2 % |
| living.situation_Lived.with.other.people | 15,3 % | 12,2 % |  | 15,7 % | 12,3 % |  | 10,6 % | 7,4 % |  | 18,8 % | 15,5 % |
| living.situation_Unknown | 5,4 % | 10,2 % |  | 3,4 % | 5,3 % |  | 8,5 % | 3,7 % |  | 3,1 % | 14,7 % |
| responsible.minors_Unknown | 4,5 % | 1,0 % |  | 1,1 % | 0,3 % |  | 10,6 % | 0,0 % |  | 0,0 % | 1,7 % |
| responsible.minors_Yes | 13,5 % | 5,1 % |  | 21,1 % | 18,1 % |  | 21,3 % | 7,4 % |  | 7,8 % | 3,4 % |
| mental.disorder_Depressive.or.anxiety.disorder | 32,4 % | 37,1 % |  | 54,8 % | 52,4 % |  | 38,3 % | 44,4 % |  | 28,1 % | 31,9 % |
| mental.disorder_Other.mental.disorders | 7,2 % | 20,3 % |  | 9,6 % | 19,2 % |  | 6,4 % | 18,5 % |  | 7,8 % | 21,6 % |
| mental.disorder_Psychosis.or.bipolar.disorder | 17,1 % | 16,2 % |  | 21,8 % | 17,5 % |  | 4,3 % | 12,3 % |  | 26,6 % | 19,0 % |
| charlson.score_X1.2 | 27,0 % | 22,3 % |  | 12,6 % | 15,6 % |  | 29,8 % | 18,5 % |  | 25,0 % | 25,0 % |
| charlson.score_X3. | 7,2 % | 6,1 % |  | 3,4 % | 5,0 % |  | 6,4 % | 7,4 % |  | 7,8 % | 5,2 % |
| duration.psychiatric.history_Less.than.1.year | 10,8 % | 6,6 % |  | 36,0 % | 22,3 % |  | 12,8 % | 6,2 % |  | 9,4 % | 6,9 % |
| duration.psychiatric.history_More.than.5.years | 58,6 % | 74,6 % |  | 34,5 % | 55,4 % |  | 48,9 % | 69,1 % |  | 65,6 % | 78,4 % |
| violence_Unknown | 14,4 % | 12,2 % |  | 5,0 % | 11,7 % |  | 17,0 % | 4,9 % |  | 12,5 % | 17,2 % |
| violence_Yes | 13,5 % | 29,4 % |  | 6,5 % | 11,1 % |  | 8,5 % | 14,8 % |  | 17,2 % | 39,7 % |
| prison_Unknown | 29,7 % | 28,4 % |  | 6,5 % | 18,4 % |  | 36,2 % | 22,2 % |  | 25,0 % | 32,8 % |
| prison_Yes | 15,3 % | 22,8 % |  | 3,8 % | 7,5 % |  | 17,0 % | 8,6 % |  | 14,1 % | 32,8 % |
| childhood.abuse_Unknown | 31,5 % | 36,0 % |  | 19,2 % | 28,1 % |  | 36,2 % | 25,9 % |  | 28,1 % | 43,1 % |
| childhood.abuse_Yes | 23,4 % | 39,6 % |  | 13,0 % | 23,7 % |  | 27,7 % | 43,2 % |  | 20,3 % | 37,1 % |
| lifevent_Unknown | 23,4 % | 25,4 % |  | 17,6 % | 21,2 % |  | 38,3 % | 22,2 % |  | 12,5 % | 27,6 % |
| lifevent_Yes | 43,2 % | 40,6 % |  | 36,0 % | 40,4 % |  | 40,4 % | 45,7 % |  | 45,3 % | 37,1 % |
| economic.problems_Unknown | 35,1 % | 38,6 % |  | 21,1 % | 24,5 % |  | 46,8 % | 34,6 % |  | 26,6 % | 41,4 % |
| economic.problems_Yes | 18,0 % | 13,7 % |  | 7,3 % | 13,9 % |  | 17,0 % | 13,6 % |  | 18,8 % | 13,8 % |
| suicide.family_Unknown | 48,6 % | 49,7 % |  | 28,0 % | 32,6 % |  | 57,4 % | 40,7 % |  | 42,2 % | 56,0 % |
| suicide.family_Yes..family | 6,3 % | 7,6 % |  | 6,9 % | 10,0 % |  | 8,5 % | 9,9 % |  | 4,7 % | 6,0 % |
| inpatient.n.cat_X1.admission | 27,0 % | 18,3 % |  | 19,9 % | 26,2 % |  | 27,7 % | 23,5 % |  | 26,6 % | 14,7 % |
| inpatient.n.cat_X2.or.more.admissions | 39,6 % | 60,9 % |  | 29,5 % | 40,4 % |  | 23,4 % | 53,1 % |  | 51,6 % | 66,4 % |
| outpatient.n.cat_More.than.5.contacts | 53,2 % | 65,0 % |  | 48,7 % | 52,1 % |  | 38,3 % | 74,1 % |  | 64,1 % | 58,6 % |
| outpatient.n.cat_No.outpatient.contact | 6,3 % | 9,6 % |  | 7,3 % | 10,6 % |  | 8,5 % | 8,6 % |  | 4,7 % | 10,3 % |
| last.admission.duration_Less.than.7.days | 32,4 % | 40,1 % |  | 15,7 % | 29,0 % |  | 27,7 % | 42,0 % |  | 35,9 % | 38,8 % |
| last.admission.duration_Not.relevant | 33,3 % | 20,8 % |  | 50,6 % | 33,4 % |  | 48,9 % | 23,5 % |  | 21,9 % | 19,0 % |
| last.admission.readmission_Not.relevant | 69,4 % | 48,2 % |  | 81,2 % | 71,3 % |  | 85,1 % | 61,7 % |  | 57,8 % | 38,8 % |
| last.admission.readmission_Yes | 10,8 % | 19,3 % |  | 7,3 % | 10,9 % |  | 6,4 % | 16,0 % |  | 14,1 % | 21,6 % |
| last.discharge_Irregular.discharge | 13,5 % | 16,8 % |  | 3,8 % | 10,0 % |  | 10,6 % | 13,6 % |  | 15,6 % | 19,0 % |
| last.discharge_No.admission | 45,9 % | 30,5 % |  | 58,6 % | 50,4 % |  | 53,2 % | 28,4 % |  | 40,6 % | 31,9 % |
| followup.discharge_More.than.one.week | 13,5 % | 12,2 % |  | 10,7 % | 15,6 % |  | 12,8 % | 13,6 % |  | 14,1 % | 11,2 % |
| followup.discharge_No.appointment | 10,8 % | 11,2 % |  | 6,1 % | 9,2 % |  | 12,8 % | 11,1 % |  | 9,4 % | 11,2 % |
| followup.discharge_Not.relevant | 39,6 % | 32,5 % |  | 56,7 % | 45,1 % |  | 44,7 % | 30,9 % |  | 35,9 % | 33,6 % |
| outpatient.status_Ended.contact | 33,3 % | 21,3 % |  | 29,1 % | 25,6 % |  | 46,8 % | 25,9 % |  | 23,4 % | 18,1 % |
| outpatient.status_Not.relevant | 9,9 % | 9,6 % |  | 11,1 % | 18,9 % |  | 4,3 % | 4,9 % |  | 14,1 % | 12,9 % |
| outpatient.status_Unknown | 7,2 % | 8,1 % |  | 5,7 % | 6,7 % |  | 8,5 % | 7,4 % |  | 6,3 % | 8,6 % |
| outpatient.unit_Acute.team | 6,3 % | 9,6 % |  | 15,7 % | 12,8 % |  | 2,1 % | 14,8 % |  | 9,4 % | 6,0 % |
| outpatient.unit_Ambulatory.team..including.ACT.FACT. | 9,0 % | 12,2 % |  | 9,2 % | 6,7 % |  | 4,3 % | 9,9 % |  | 12,5 % | 13,8 % |
| outpatient.unit_Not.relevant | 9,9 % | 9,6 % |  | 11,1 % | 18,9 % |  | 4,3 % | 4,9 % |  | 14,1 % | 12,9 % |
| outpatient.unit_Other | 20,7 % | 13,7 % |  | 16,5 % | 16,2 % |  | 17,0 % | 12,3 % |  | 23,4 % | 14,7 % |
| outpatient.unit_Unknown | 3,6 % | 6,6 % |  | 4,2 % | 5,6 % |  | 4,3 % | 8,6 % |  | 3,1 % | 5,2 % |
| outpatient.consult_Emergency.care.consultation | 8,1 % | 7,6 % |  | 7,3 % | 8,1 % |  | 6,4 % | 9,9 % |  | 9,4 % | 6,0 % |
| outpatient.consult_Not.relevant | 9,9 % | 9,6 % |  | 11,1 % | 18,9 % |  | 4,3 % | 4,9 % |  | 14,1 % | 12,9 % |
| outpatient.consult_Other | 18,0 % | 20,8 % |  | 18,0 % | 10,9 % |  | 19,1 % | 14,8 % |  | 17,2 % | 25,0 % |
| outpatient.consult_Unknown | 3,6 % | 8,1 % |  | 2,3 % | 5,6 % |  | 4,3 % | 7,4 % |  | 3,1 % | 8,6 % |
| primary.care_Unknown | 20,7 % | 16,2 % |  | 11,1 % | 15,3 % |  | 25,5 % | 16,0 % |  | 17,2 % | 16,4 % |
| primary.care_Yes | 41,4 % | 53,3 % |  | 25,3 % | 36,5 % |  | 31,9 % | 45,7 % |  | 48,4 % | 58,6 % |
| individual.plan_Offered.but.did.not.want | 9,0 % | 9,1 % |  | 5,0 % | 6,4 % |  | 4,3 % | 6,2 % |  | 12,5 % | 11,2 % |
| individual.plan_Unknown | 14,4 % | 24,4 % |  | 7,3 % | 13,4 % |  | 14,9 % | 19,8 % |  | 14,1 % | 27,6 % |
| individual.plan_Yes | 6,3 % | 7,6 % |  | 3,4 % | 4,7 % |  | 2,1 % | 4,9 % |  | 9,4 % | 9,5 % |
| antipsychotics.per.os_Prescribed | 22,5 % | 35,5 % |  | 32,6 % | 36,5 % |  | 14,9 % | 29,6 % |  | 28,1 % | 39,7 % |
| antipsychotics.per.os_Unknown | 9,9 % | 12,2 % |  | 4,6 % | 7,8 % |  | 17,0 % | 12,3 % |  | 4,7 % | 12,1 % |
| antipsychotics.depot_Prescribed | 6,3 % | 6,1 % |  | 8,8 % | 5,3 % |  | 0,0 % | 2,5 % |  | 10,9 % | 8,6 % |
| antipsychotics.depot_Unknown | 9,9 % | 10,7 % |  | 5,0 % | 8,1 % |  | 17,0 % | 9,9 % |  | 4,7 % | 11,2 % |
| moodstabilizer_Prescribed | 5,4 % | 9,1 % |  | 8,8 % | 12,8 % |  | 2,1 % | 9,9 % |  | 7,8 % | 8,6 % |
| moodstabilizer_Unknown | 9,9 % | 10,2 % |  | 4,2 % | 8,1 % |  | 17,0 % | 11,1 % |  | 4,7 % | 9,5 % |
| antidepressants.tca_Prescribed | 0,0 % | 7,1 % |  | 8,8 % | 6,7 % |  | 0,0 % | 6,2 % |  | 0,0 % | 7,8 % |
| antidepressants.tca_Unknown | 10,8 % | 11,7 % |  | 4,2 % | 9,7 % |  | 19,1 % | 13,6 % |  | 4,7 % | 10,3 % |
| antidepressants.other_Prescribed | 21,6 % | 21,8 % |  | 37,9 % | 45,4 % |  | 25,5 % | 32,1 % |  | 18,8 % | 14,7 % |
| antidepressants.other_Unknown | 9,0 % | 13,2 % |  | 4,6 % | 9,5 % |  | 14,9 % | 13,6 % |  | 4,7 % | 12,9 % |
| benzodiazepines_Prescribed | 28,8 % | 31,0 % |  | 26,4 % | 35,9 % |  | 14,9 % | 29,6 % |  | 39,1 % | 31,9 % |
| benzodiazepines_Unknown | 11,7 % | 14,2 % |  | 5,4 % | 10,0 % |  | 19,1 % | 16,0 % |  | 6,3 % | 12,9 % |
| hypnotics_Prescribed | 18,9 % | 28,4 % |  | 29,1 % | 39,3 % |  | 14,9 % | 27,2 % |  | 21,9 % | 29,3 % |
| hypnotics_Unknown | 14,4 % | 13,7 % |  | 6,9 % | 11,1 % |  | 19,1 % | 14,8 % |  | 10,9 % | 12,9 % |
| opiates.other_Unknown | 9,9 % | 9,1 % |  | 5,0 % | 9,7 % |  | 14,9 % | 9,9 % |  | 6,3 % | 8,6 % |
| opiates.substitution_Unknown | 9,0 % | 8,6 % |  | 5,4 % | 8,9 % |  | 14,9 % | 8,6 % |  | 4,7 % | 8,6 % |
| medication.other_Prescribed | 12,6 % | 10,2 % |  | 8,0 % | 10,6 % |  | 17,0 % | 11,1 % |  | 9,4 % | 9,5 % |
| medication.other_Unknown | 13,5 % | 14,7 % |  | 7,7 % | 13,9 % |  | 19,1 % | 16,0 % |  | 9,4 % | 13,8 % |
| medication.not.wanted_Not.relevant | 18,0 % | 16,8 % |  | 21,5 % | 19,5 % |  | 4,3 % | 13,6 % |  | 28,1 % | 19,0 % |
| medication.not.wanted_Unknown | 15,3 % | 22,8 % |  | 8,0 % | 16,4 % |  | 27,7 % | 21,0 % |  | 6,3 % | 24,1 % |
| medication.not.wanted_Yes | 4,5 % | 7,1 % |  | 10,7 % | 8,6 % |  | 0,0 % | 7,4 % |  | 7,8 % | 6,9 % |
| medication.compliance_No | 5,4 % | 6,6 % |  | 5,4 % | 8,6 % |  | 0,0 % | 4,9 % |  | 9,4 % | 7,8 % |
| medication.compliance_No.prescription | 13,5 % | 11,7 % |  | 14,2 % | 13,4 % |  | 12,8 % | 12,3 % |  | 14,1 % | 11,2 % |
| medication.compliance_Unknown | 52,3 % | 49,2 % |  | 39,1 % | 38,7 % |  | 74,5 % | 59,3 % |  | 35,9 % | 42,2 % |
| medication.sideeffects_No.prescription | 23,4 % | 16,2 % |  | 15,3 % | 12,8 % |  | 29,8 % | 21,0 % |  | 18,8 % | 12,9 % |
| medication.sideeffects_Unknown | 23,4 % | 28,4 % |  | 17,2 % | 23,4 % |  | 34,0 % | 21,0 % |  | 15,6 % | 33,6 % |
| medication.sideeffects_Yes | 7,2 % | 7,1 % |  | 13,8 % | 14,2 % |  | 2,1 % | 7,4 % |  | 10,9 % | 6,9 % |
| last.contact.levelofcare_Inpatient | 35,1 % | 44,2 % |  | 24,1 % | 38,4 % |  | 25,5 % | 39,5 % |  | 42,2 % | 47,4 % |
| nonattendance_Not.relevant | 12,6 % | 13,7 % |  | 8,8 % | 10,3 % |  | 10,6 % | 9,9 % |  | 14,1 % | 16,4 % |
| nonattendance_Yes | 27,9 % | 29,4 % |  | 18,4 % | 24,0 % |  | 36,2 % | 35,8 % |  | 21,9 % | 25,0 % |
| last.contact.type_Phone | 26,1 % | 21,8 % |  | 18,8 % | 14,8 % |  | 36,2 % | 28,4 % |  | 18,8 % | 17,2 % |
| last.contact.profession_MD.under.specialization | 10,8 % | 9,6 % |  | 8,4 % | 9,7 % |  | 8,5 % | 3,7 % |  | 12,5 % | 13,8 % |
| last.contact.profession_Nurse | 18,0 % | 21,3 % |  | 19,9 % | 20,9 % |  | 19,1 % | 16,0 % |  | 17,2 % | 25,0 % |
| last.contact.profession_Other | 30,6 % | 25,4 % |  | 15,7 % | 18,9 % |  | 31,9 % | 29,6 % |  | 29,7 % | 22,4 % |
| last.contact.profession_Psychologist | 16,2 % | 12,2 % |  | 13,0 % | 14,5 % |  | 19,1 % | 14,8 % |  | 14,1 % | 10,3 % |
| last.contact.profession_Specialist.in.clinical.psychology | 6,3 % | 11,2 % |  | 15,7 % | 11,7 % |  | 8,5 % | 12,3 % |  | 4,7 % | 10,3 % |
| last.contact.primary.contact_No | 15,3 % | 21,3 % |  | 16,1 % | 21,2 % |  | 14,9 % | 21,0 % |  | 15,6 % | 21,6 % |
| last.contact.primary.contact_No.primary.contact | 8,1 % | 7,1 % |  | 10,3 % | 8,4 % |  | 4,3 % | 8,6 % |  | 10,9 % | 6,0 % |
| crisisplan_No | 59,5 % | 48,7 % |  | 64,4 % | 53,5 % |  | 66,0 % | 49,4 % |  | 54,7 % | 48,3 % |
| crisisplan_Unknown | 13,5 % | 22,8 % |  | 9,6 % | 15,9 % |  | 14,9 % | 18,5 % |  | 12,5 % | 25,9 % |
| relatives.informed_No | 20,7 % | 13,7 % |  | 7,7 % | 10,9 % |  | 29,8 % | 13,6 % |  | 14,1 % | 13,8 % |
| relatives.informed_Not.relevant | 44,1 % | 48,7 % |  | 57,1 % | 52,4 % |  | 40,4 % | 49,4 % |  | 46,9 % | 48,3 % |
| relatives.informed_Unknown | 14,4 % | 17,3 % |  | 12,6 % | 12,5 % |  | 10,6 % | 12,3 % |  | 17,2 % | 20,7 % |
| anxiety.symptoms_Unknown | 10,8 % | 16,8 % |  | 6,5 % | 9,2 % |  | 14,9 % | 13,6 % |  | 7,8 % | 19,0 % |
| anxiety.symptoms_Yes | 30,6 % | 40,1 % |  | 40,6 % | 41,5 % |  | 23,4 % | 42,0 % |  | 35,9 % | 38,8 % |
| depressive.symptoms_Unknown | 9,9 % | 15,7 % |  | 5,4 % | 6,7 % |  | 12,8 % | 9,9 % |  | 7,8 % | 19,8 % |
| depressive.symptoms_Yes | 31,5 % | 40,6 % |  | 50,2 % | 50,1 % |  | 25,5 % | 49,4 % |  | 35,9 % | 34,5 % |
| deterioration.physical_Unknown | 17,1 % | 18,8 % |  | 10,3 % | 12,3 % |  | 21,3 % | 16,0 % |  | 14,1 % | 20,7 % |
| deterioration.physical_Yes | 9,0 % | 8,1 % |  | 11,9 % | 12,8 % |  | 8,5 % | 7,4 % |  | 9,4 % | 8,6 % |
| psychotic.symptoms_Unknown | 12,6 % | 11,2 % |  | 5,4 % | 6,4 % |  | 10,6 % | 8,6 % |  | 14,1 % | 12,9 % |
| psychotic.symptoms_Yes | 10,8 % | 8,1 % |  | 8,0 % | 5,8 % |  | 2,1 % | 3,7 % |  | 17,2 % | 11,2 % |
| hostility_Unknown | 7,2 % | 10,7 % |  | 4,2 % | 5,3 % |  | 8,5 % | 3,7 % |  | 6,3 % | 15,5 % |
| hostility_Yes | 5,4 % | 10,2 % |  | 3,8 % | 4,2 % |  | 2,1 % | 8,6 % |  | 7,8 % | 11,2 % |
| increased.alcohol.use_Unknown | 18,0 % | 20,3 % |  | 13,0 % | 14,5 % |  | 21,3 % | 16,0 % |  | 15,6 % | 23,3 % |
| increased.alcohol.use_Yes | 14,4 % | 18,3 % |  | 2,3 % | 5,3 % |  | 25,5 % | 34,6 % |  | 6,3 % | 6,9 % |
| increased.drug.use_Unknown | 22,5 % | 23,4 % |  | 10,7 % | 16,4 % |  | 25,5 % | 22,2 % |  | 20,3 % | 24,1 % |
| increased.drug.use_Yes | 18,0 % | 18,3 % |  | 1,9 % | 2,8 % |  | 8,5 % | 8,6 % |  | 25,0 % | 25,0 % |
| hopelessness_Unknown | 16,2 % | 16,8 % |  | 8,8 % | 12,3 % |  | 21,3 % | 13,6 % |  | 12,5 % | 19,0 % |
| hopelessness_Yes | 14,4 % | 34,5 % |  | 30,7 % | 30,9 % |  | 6,4 % | 42,0 % |  | 20,3 % | 29,3 % |
| suicidal.ideation_Unknown | 10,8 % | 17,8 % |  | 11,9 % | 9,7 % |  | 12,8 % | 16,0 % |  | 9,4 % | 19,0 % |
| suicidal.ideation_Yes | 6,3 % | 28,4 % |  | 19,2 % | 32,9 % |  | 6,4 % | 27,2 % |  | 6,3 % | 29,3 % |
| sleep.problems_Unknown | 20,7 % | 32,0 % |  | 16,9 % | 20,6 % |  | 25,5 % | 37,0 % |  | 17,2 % | 28,4 % |
| sleep.problems_Yes | 24,3 % | 24,9 % |  | 31,8 % | 29,2 % |  | 19,1 % | 23,5 % |  | 28,1 % | 25,9 % |

# **Supplementary material 4.** *Cross-validations curves*

# The cross-validation curves with confidence intervals over the regularization parameter Lambda in patients with substance use (A1) or no substance use disorders (A2) and patients with alcohol (B1) or drug use disorders (B2). The vertical lines indicates the lambda values for the highest AUC and the 1 standard error below highest AUC (used here).


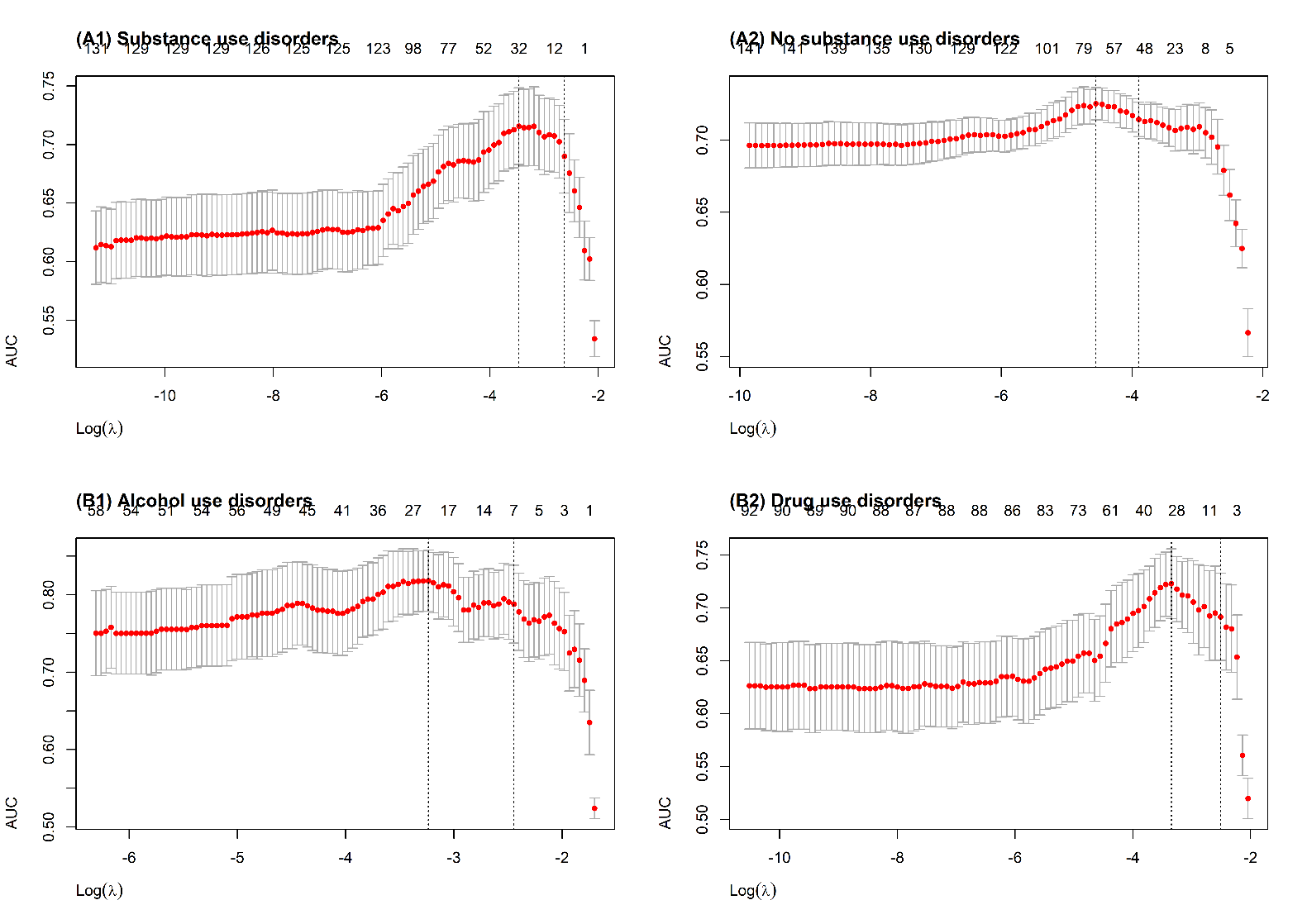


# **Supplementary material 5.** ROC curves illustrating the true positive and true negative rates.


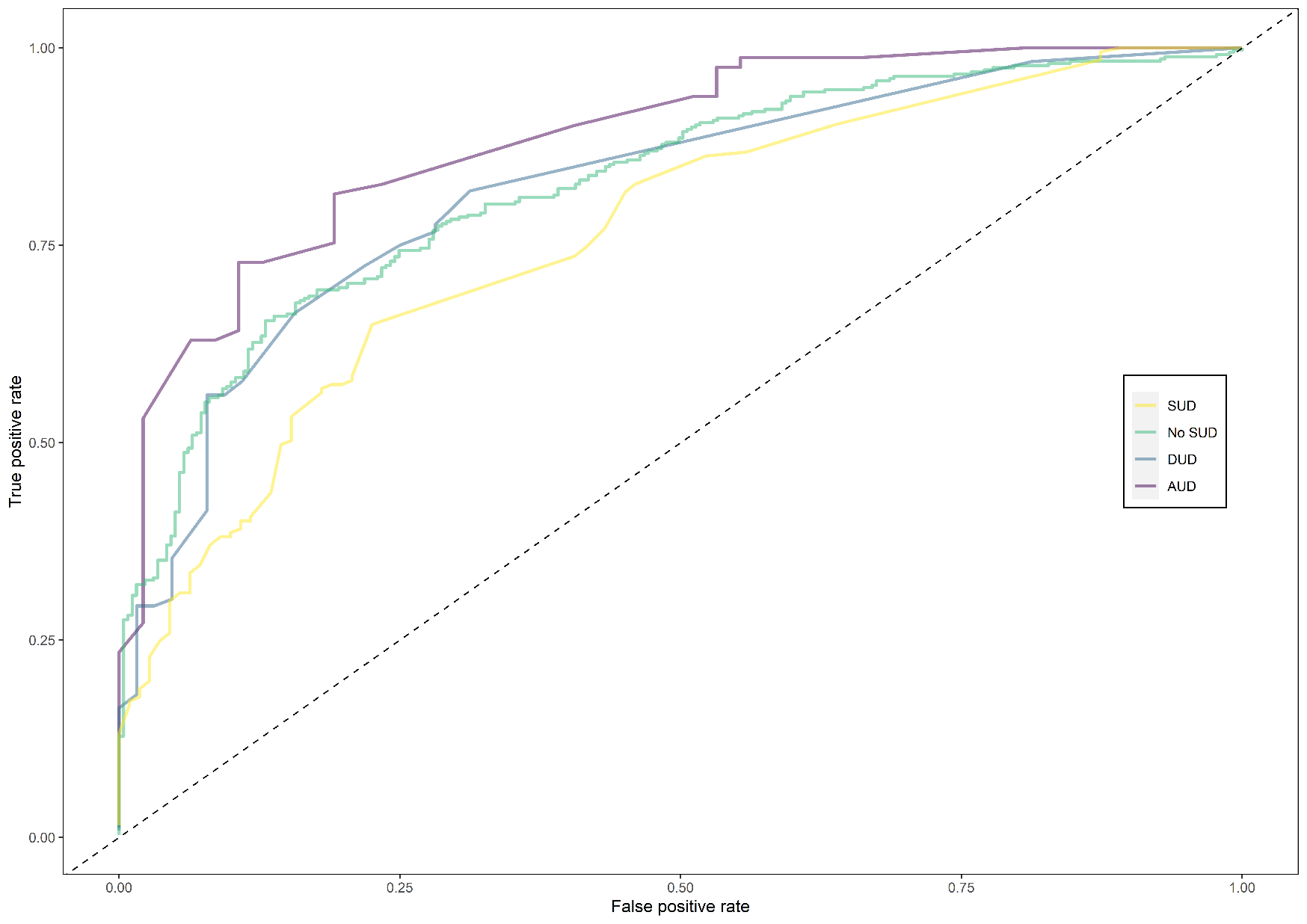


# **Supplementary material 6.** The association between the factors and self-harm in factors selected by LASSO regression with conditional post-selection 90 % confidence intervals in patients with and without substance use disorders who died by suicide.

|  | Patients with SUDs | | | |  | Patients without SUDs | | | |
| --- | --- | --- | --- | --- | --- | --- | --- | --- | --- |
| Factors | LASSO  OR | Post-selection  90 % LCI | Post-selection  90 % UCI | *p* |  | LASSO  OR | Post-selection  90 % LCI | Post-selection  90 % UCI | *p* |
| personality.disorders | 1.96 | 1.12 | 3.40 | **0.047** |  | 2.29 | 1.01 | 5.18 | 0.095 |
| income_Other | 0.37 | 0.22 | 0.60 | **0.001** |  | 0 | - | - | - |
| violence_Yes | 1.86 | 1.20 | 2.87 | **0.019** |  | 1.23 | 0.62 | 2.46 | 0.623 |
| childhood.abuse_Yes | 1.44 | 0.98 | 2.11 | 0.123 |  | 1.24 | 0.74 | 2.07 | 0.493 |
| inpatient.n.cat_X2.or.more.admissions | 1.45 | 1.04 | 2.00 | **0.062** |  | 0 | - | - | - |
| hopelessness_Yes | 1.46 | 0.92 | 2.31 | 0.184 |  | 0 | - | - | - |
| suicidal.ideation_Yes | 2.98 | 1.74 | 5.10 | **0.001** |  | 2.21 | 1.44 | 3.41 | **0.002** |
| affective.disorders | 0 | - | - | - |  | 0.93 | 0.66 | 1.31 | 0.714 |
| gender_Woman | 0 | - | - | - |  | 2.05 | 1.42 | 2.97 | **0.001** |
| age_18.31 | 0 | - | - | - |  | 1.08 | 0.68 | 1.70 | 0.785 |
| age_57.88 | 0 | - | - | - |  | 0.55 | 0.37 | 0.83 | **0.015** |
| region_West | 0 | - | - | - |  | 1.43 | 0.92 | 2.21 | 0.183 |
| housing_Other.arrangements | 0 | - | - | - |  | 1.43 | 0.71 | 2.88 | 0.398 |
| housing_Unknown | 0 | - | - | - |  | 1.15 | 0.56 | 2.34 | 0.758 |
| living.situation_Lived.alone | 0 | - | - | - |  | 1.02 | 0.72 | 1.45 | 0.915 |
| mental.disorder_Other.mental.disorders | 0 | - | - | - |  | 1.15 | 0.61 | 2.16 | 0.723 |
| mental.disorder_Psychosis.or.bipolar.disorder | 0 | - | - | - |  | 0.55 | 0.34 | 0.90 | **0.047** |
| duration.psychiatric.history_More.than.5.years | 0 | - | - | - |  | 1.75 | 1.18 | 2.57 | **0.018** |
| violence_Unknown | 0 | - | - | - |  | 1.80 | 0.86 | 3.75 | 0.193 |
| prison_Unknown | 0 | - | - | - |  | 1.98 | 1.02 | 3.86 | 0.091 |
| prison_Yes | 0 | - | - | - |  | 2.60 | 1.09 | 6.15 | 0.069 |
| childhood.abuse_Unknown | 0 | - | - | - |  | 1.25 | 0.79 | 1.97 | 0.424 |
| economic.problems_Yes | 0 | - | - | - |  | 1.99 | 1.08 | 3.65 | **0.064** |
| last.admission.duration_Less.than.7.days | 0 | - | - | - |  | 0.97 | 0.59 | 1.59 | 0.920 |
| last.admission.duration_Not.relevant | 0 | - | - | - |  | 0.71 | 0.42 | 1.20 | 0.282 |
| last.discharge_Irregular.discharge | 0 | - | - | - |  | 1.53 | 0.68 | 3.43 | 0.389 |
| followup.discharge_More.than.one.week | 0 | - | - | - |  | 1.01 | 0.58 | 1.74 | 0.986 |
| followup.discharge_Not.relevant | 0 | - | - | - |  | 0.38 | 0.23 | 0.63 | **0.002** |
| outpatient.status_Not.relevant | 0 | - | - | - |  | 1.95 | 1.28 | 2.96 | **0.009** |
| outpatient.unit_Not.relevant | 0 | - | - | - |  | 1.77 | 1.17 | 2.68 | **0.024** |
| outpatient.consult_Not.relevant | 0 | - | - | - |  | 2.01 | 1.16 | 348 | **0.037** |
| outpatient.consult_Other | 0 | - | - | - |  | 0.45 | 0.27 | 0.77 | **0.014** |
| outpatient.consult_Unknown | 0 | - | - | - |  | 2.27 | 0.78 | 6.59 | 0.208 |
| primary.care_Yes | 0 | - | - | - |  | 1.04 | 0.67 | 1.60 | 0.893 |
| antipsychotics.depot_Prescribed | 0 | - | - | - |  | 0.38 | 0.18 | 0.81 | **0.035** |
| antidepressants.tca_Prescribed | 0 | - | - | - |  | 0.44 | 0.23 | 0.84 | **0.038** |
| antidepressants.other_Prescribed | 0 | - | - | - |  | 1.05 | 0.72 | 1.55 | 0.825 |
| antidepressants.other_Unknown | 0 | - | - | - |  | 1.55 | 0.58 | 4.16 | 0.469 |
| benzodiazepines_Prescribed | 0 | - | - | - |  | 1.20 | 0.79 | 1.84 | 0.475 |
| benzodiazepines_Unknown | 0 | - | - | - |  | 1.41 | 0.55 | 3.58 | 0.553 |
| hypnotics_Prescribed | 0 | - | - | - |  | 1.48 | 0.97 | 2.24 | 0.126 |
| medication.other_Unknown | 0 | - | - | - |  | 1.33 | 0.60 | 2.95 | 0.563 |
| medication.not.wanted_Unknown | 0 | - | - | - |  | 1.43 | 0.79 | 2.59 | 0.328 |
| medication.compliance_No | 0 | - | - | - |  | 1.45 | 0.72 | 2.92 | 0.380 |
| last.contact.levelofcare_Inpatient | 0 | - | - | - |  | 0.92 | 0.52 | 1.64 | 0.828 |
| nonattendance_Yes | 0 | - | - | - |  | 1.03 | 0.66 | 1.60 | 0.913 |
| last.contact.type_Phone | 0 | - | - | - |  | 0.47 | 0.29 | 0.77 | **0.012** |
| last.contact.profession_Psychologist | 0 | - | - | - |  | 1.04 | 0.63 | 1.72 | 0.896 |
| last.contact.profession_Specialist.in.clinical.psychology | 0 | - | - | - |  | 0.47 | 0.29 | 0.78 | **0.014** |
| relatives.informed_No | 0 | - | - | - |  | 1.37 | 0.74 | 2.51 | 0.400 |
| psychotic.symptoms_Yes | 0 | - | - | - |  | 0.56 | 0.27 | 1.16 | 0.190 |
| sleep.problems_Yes | 0 | - | - | - |  | 0.51 | 0.35 | 0.76 | **0.005** |

***Note.*** Factors with a p value < 10 % illustrated with bold text.

# **Supplementary 7**. The association between the factors and self-harm in factors selected by LASSO regression with conditional post-selection 90 % confidence intervals in patients with alcohol og drug use disorders who died by suicide.

|  | Patients with Alcohol Use Disorders | | | |  | Patients with Drug Use Disorders | | | |
| --- | --- | --- | --- | --- | --- | --- | --- | --- | --- |
| Factors | LASSO  OR | Post-selection  90 % LCI | Post-selection  90 % UCI | *p* |  | LASSO  OR | Post-selection  90 % LCI | Post-selection  90 % UCI | *p* |
| gender_Woman | 1.98 | 0.96 | 4.02 | 0.118 |  | 0 | - | - | - |
| responsible.minors_Unknown | 0.09 | 0.02 | 0.39 | **0.007** |  | 0 | - | - | - |
| inpatient.n.cat_X2.or.more.admissions | 1.62 | 0.87 | 2.97 | 0.201 |  | 0 | - | - | - |
| outpatient.n.cat_More.than.5.contacts | 2.51 | 1.35 | 4.66 | **0.014** |  | 0 | - | - | - |
| last.discharge_No.admission | 0.26 | 0.14 | 0.48 | **0.000** |  | 0 | - | - | - |
| outpatient.unit_Acute.team | 5.12 | 1.73 | 15.03 | **0.013** |  | 0 | - | - | - |
| hopelessness_Yes | 4.84 | 2.18 | 10.69 | **0.001** |  | 0 | - | - | - |
| personality.disorders | 0 | - | - | - |  | 2.91 | 1.44 | 5.89 | **0.013** |
| income_Other | 0 | - | - | - |  | 0.35 | 0.18 | 0.67 | **0.008** |
| income_Unknown | 0 | - | - | - |  | 1.94 | 0.81 | 4.61 | 0.218 |
| violence_Yes | 0 | - | - | - |  | 1.98 | 1.10 | 3.53 | **0.054** |
| prison_Yes | 0 | - | - | - |  | 1.37 | 0.72 | 2.58 | 0.424 |
| medication.not.wanted_Unknown | 0 | - | - | - |  | 2.06 | 1.02 | 4.15 | **0.093** |
| suicidal.ideation_Yes | 0 | - | - | - |  | 3.59 | 1.96 | 6.60 | **0.001** |

***Note.*** Factors with a p value < 10 % illustrated with bold text.
